# Short Form of the Pediatric Symptom Checklist-Youth Self-Report (PSC-17-Y): Spanish Validation Study

**DOI:** 10.2196/31127

**Published:** 2021-12-01

**Authors:** Jose A Piqueras, Verónica Vidal-Arenas, Raquel Falcó, Beatriz Moreno-Amador, Juan C Marzo, Juliana M Holcomb, Michael Murphy

**Affiliations:** 1 Area of Personality, Assessment and Psychological Treatment Department of Health Psychology Universidad Miguel Hernandez de Elche Elche Spain; 2 Department of Basic and Clinical Psychology and Psychobiology Universitat Jaume I Castellon Spain; 3 Area of Social Psychology Department of Health Psychology Universidad Miguel Hernandez de Elche Elche Spain; 4 Department of Psychiatry Massachusetts General Hospital Boston, MA United States; 5 Department of Psychiatry Harvard Medical School Boston, MA United States

**Keywords:** PSC-17-Y, psychometric properties, screening, mental problems, adolescents, adolescent health, adolescent medicine, psychiatry, psychology, psychosocial issues

## Abstract

**Background:**

The short form, 17-item version of the Pediatric Symptom Checklist-Youth Self-Report (PSC-17-Y) is a validated measure that assesses psychosocial problems overall (OVR) and in 3 major psychopathological domains (internalizing, externalizing, and attention-deficit/hyperactivity disorder), taking 5-10 min to complete. Prior research has established sound psychometric properties of the PSC-17-Y for English speakers.

**Objective:**

This study extends psychometric evidence for the acceptability of the PSC-17-Y in a large sample of Spanish adolescents, providing proof of its reliability and structure, convergent and discriminant validity, and longitudinal and gender invariance.

**Methods:**

Data were collected on 5430 adolescents, aged 12-18 years, who filled out the PSC-17-Y twice during 2018-2019 (7-month interval). We calculated the Cronbach alpha and the McDonald omega coefficients to test reliability, the Pearson correlation for convergent (distress) and criterion validity (well-being, quality of life, and socioemotional skills), confirmatory factor analysis (CFA) for structure validity, and multigroup and longitudinal measurement invariance analysis for longitudinal and gender stability.

**Results:**

Within structural analysis for the PSC-17-Y, CFA supported a correlated 3-factor solution, which was also invariant longitudinally and across gender. All 3 subscales showed evidence of reliability, with coefficients near or above .70. Moreover, scores of PSC-17-Y subscales were positively related with convergent measures and negatively related with criterion measures. Normative data for the PSC-17-Y are presented in the form of percentiles (75th and 90th).

**Conclusions:**

This work provides the first evidence of the reliability and validity of the Spanish version of the PSC-17-Y administered over the internet to assess mental health problems among adolescents, maintaining the same domains as the long version.

## Introduction

According to Polanczyk et al [1], the most common mental health disorders among children and adolescents include anxiety or depression, behavioral disorders, and attention-deficit/hyperactivity disorder (ADHD). Emotional and behavioral symptoms at the subclinical level raise the risk of subsequent development of mental disorders [2]. Moreover, the COVID-19 pandemic has provoked a considerable increase in mental health problems among children and adolescents [3-5].

National and international policies and strategies globally recommend that young people attending primary care should be routinely screened for psychosocial problems [6]. Despite this, such screening occurs in less than 50% of primary care visits of adolescents, meaning that more than half of adolescent mental health problems go undetected [7,8]. Although several screening tools exist for psychosocial problems in young people, most cover a single domain [9] and can be time consuming to administer and interpret [10]. Primary care clinicians can often be unsure of which screening tools are appropriate for their clinical context. In addition, many tools rely on the provider having the skills, knowledge, expertise, and experience to initiate screening, interpret results, and provide appropriate interventions [8]. Providers often describe a lack of resources in terms of the availability of time, appropriate tools, training, and experience in youth health [11].

A recent review of “Self-Report Rating Scales to Guide Measurement-Based Care in Child and Adolescent Psychiatry” [12] highlights that the Pediatric Symptom Checklist (PSC) is 1 of the most widely used measures to screen psychosocial problems in primary care units and school settings. This statement is supported for all parent and youth reports and for long and short forms (parent- and youth-reported long form [PSC-35]; parent- and youth-reported short form [PSC-17]) [13-23].

The short form, 17-item version of the PSC-Y (PSC-17-Y) [16] is used to assess self-reported general psychosocial functioning among youth above 11 years old, taking only 5-10 min to be completed, and is statistically equivalent to the short form of the parent version (PSC-17) [16] and to the longer youth report form (PSC-35-Y [19-21]).

Three studies of the parent report PSC-17 (Gardner et al [16,17] and Murphy et al [18]) have confirmed the existence of the 3 original subscales for internalizing (INT) symptoms, externalizing (EXT) symptoms, and ADHD symptoms (ATT) and provided evidence of the reliability of the overall (OVR) scale. Two studies with the youth-reported short form have been published. On the one hand, Bergman et al [22] found that the PSC-17-Y is equivalent to the parent-reported form of the PSC-17, indicating that a 3-factor short form with 17 items meets the criteria for scalar invariance across gender. On the other hand, Parker et al [23] examined the screening validity of the PSC-17-Y in a child welfare population. Youth with any lifetime mental health diagnosis scored significantly higher on the PSC ATT and INT subscales. The ATT, INT, and OVR subscale scores were significantly correlated with psychosis, depression, and anxiety disorder scores. ADHD was associated with ATT, OVR, and EXT scores. Only bipolar disorder was weakly associated with PSC subscale scores (EXT and OVR). This study provides support for the convergent and discriminant validity of the PSC-17-Y.

Despite PSC-17-Y’s potential, however, there is limited evidence of some of its relevant psychometric properties (eg, longitudinal measurement invariance and other reliability coefficients different from the Cronbach alpha [*α*]), and to the best of our knowledge from the scientific literature review, none of these psychometric analyses are in languages other than English (ie, none on Spanish populations).

Thus, this work aimed to extend the psychometric evidence for the acceptability of the PSC-17-Y in a large sample of Spanish adolescents, providing different sources of reliability and validity. This research could facilitate the use of the PSC -17-Y in more contexts and for more possible applications in youth mental health settings. Overall, we expected that the PSC-17-Y would show that it is a valid and reliable ultrabrief screening measure that can be administered over the internet to detect mental health problems in Spanish adolescents.

## Methods

### Sample

The final sample consisted of 5430 adolescents (2769 [51%] females) at time 0 and 2117 (1109 [52.4%] females) at time 1 (approximately 7 months later). The participants were enrolled in Spanish secondary education grades, equivalent to US middle and high school, from grades 7 (12-13 years) to 12 (17-18 years). The average age of the sample at time 0 was 14.17 years (SD 1.50) and of the sample that participated at time 0 and time 1 was 13.99 years (SD 1.39).

### Measures

#### PSC-17-Y

The PSC-17-Y [16] consists of 17 items and 3 factors to assess 3 types of problems: INT symptoms (ie, depression and anxiety), EXT symptoms (ie, disruptive behavior), and ATT, as well as an OVR score. The Spanish version of the PSC-17-Y was developed in accordance with the guidelines of the International Test Commission [24], using an iterative translation method that began with several independent translations. The item translations were then reviewed by a joint committee comprising translators with knowledge of the Spanish language and culture and specialists in the field of assessment who analyzed the adequacy of the adapted version. To be sure that adolescents properly understood all items, interviews asking about the comprehension of the items were conducted. In addition, in 2018, we had conversations with colleagues who had worked on the translation of the PSC for parents in Chile [25,26] in order to obtain an adequate cross-cultural adaptation into the European Spanish language of the PSC-17-Y.

#### Social-Emotional Distress Survey-Secondary

The Social-Emotional Distress Survey-Secondary (SEDS-S) [27] is a 10-item behavioral screening questionnaire designed to measure INT distress. The reliability of the 1-factor total scale was *α*=.91. In their study, Dowdy et al [27] found a significant positive association of the SEDS-S distress factor with symptoms of anxiety and depression and a significant negative association with life satisfaction and strength scores.

#### Mental Health Continuum-Short Form

The Mental Health Continuum-Short Form (MHC-SF) [28] is the reduced version of the MHC Long Form. This measure provides self-reported well-being, divided into 3 subfactors: psychological (6 items), emotional (3 items) and social well-being (5 items). In this study, we used the Spanish version of the MHC-SF recently adapted by our team. The MHC-SF has received psychometric support for use with adolescents across many different countries, including Spain [29,30], showing excellent internal consistency (Cronbach *α*>.80) and discriminant validity in adolescents.

#### KIDSCREEN-10 Index

The KIDSCREEN-10 Index [30] is a unidimensional scale that measures health-related quality of life (HRQoL) in healthy and chronically ill children and adolescents. It was developed to specifically identify children at risk in terms of subjective health and suggest appropriate early interventions. The instrument provides an overall HRQoL index covering the physical, psychological, and social facets of the HRQoL. Internal consistency values (Cronbach *α*) reach .82, and test-retest reliability within 2 weeks reaches .55 [31].

#### Social-Emotional Health Survey-Secondary

The Social-Emotional Health Survey-Secondary (SEHS-S) [32] was developed to measure the components of the covitality latent construct among youth. We used the Spanish version of the SEHS-S, which is appropriate for adolescents aged 12-18 years [33]. The SEHS-S includes 36 items for the assessment of core psychosocial assets based on a higher-order model comprising 12 first-order, grouped into 4 second-order, latent traits (3 each) and a higher-order general factor (covitality). The first domain, called belief-in-self, measures self-efficacy, self-awareness, and persistence. The domain belief-in-others comprises school support, peer support, and family support. The domain emotional competence considers emotion regulation, empathy, and behavioral self-control. Engaged living, which is the final domain, comprises 3 subscales: gratitude, zest, and optimism.

### Procedure

This research used a non-experimental, transversal/longitudinal, quantitative, and descriptive-correlational design [34,35]. The UMH Project Evaluation Committee approved the study (reference no. DPS.JPR.02.17). Once the project was approved, quota sampling was carried out in 2 areas of southeastern Spain: the province of Alicante (PA) belonging to the Valencian Community, and the Autonomous Community of the Region of Murcia (RM), making a random selection of secondary schools based on ownership (public/nonpublic schools) and regional geographical areas (9 areas in PA and 21 in RM). After 100 schools were contacted, 13 from PA and 21 from RM agreed to participate, resulting in a total of 34 secondary schools (22 [65.2%] public and 12 [34.8%] nonpublic schools; 30 [87%] secular and 4 [13%] catholic schools).

Once the schools agreed to participate, signed informed consent was requested in writing from the parents/legal guardians of the adolescent participants and from the adolescents themselves, accepting participation in the research. The data collection was carried out in the schools and supervised by the research staff in person. The self-reporting assessment protocol was individually applied through the online survey tool LimeSurvey (LimeSurvey GmbH, Hamburg, Germany). Participation was voluntary, and the adolescents did not receive any incentive for their collaboration, while each school received a feedback report, including results by class group.

### Data Analysis

**Figure 1:**
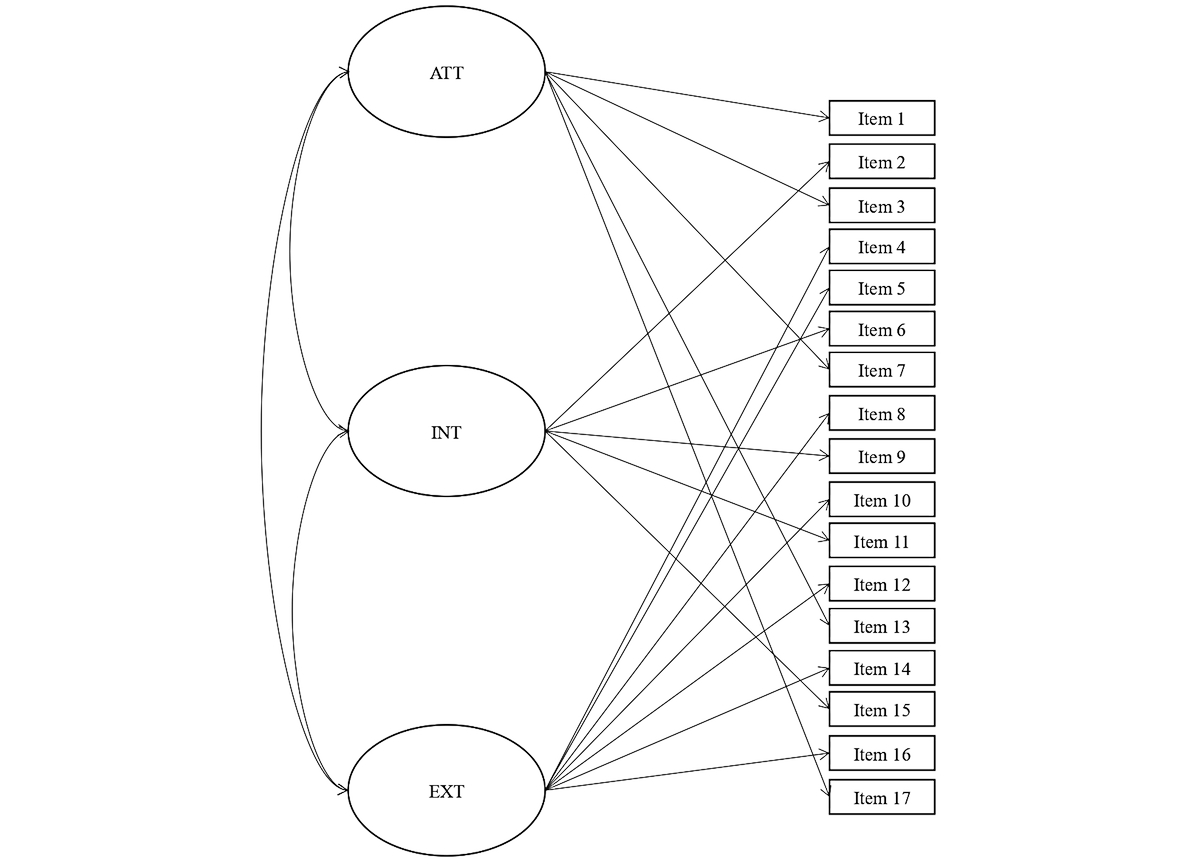
Visual representation of the 17-item, 3-factor solution of the PSC-17-Y. ATT: attention-deficit/hyperactivity disorder symptoms; EXT: externalizing; INT: internalizing; PSC-17-Y: Pediatric Symptom Checklist-Youth Self-Report.

All analyses were conducted using IBM SPSS Statistics version 25 and Mplus 8.4 (Muthén & Muthén). Confirmatory factor analysis (CFA) was conducted to test the structural validity. Figure 1 represents the correlated 3-factor solution tested. We used a diagonally weighted least squares means and variance adjusted (WLSMV) model estimator due to a number of alternative responses and the nonnormality distribution [36]. We tested the model’s goodness of fit using the comparative fit index (CFI), the Tucker-Lewis index (TLI), and the root-mean-square error of approximation (RMSEA). A CFI of >0.90 and a TLI of >0.95 indicate an acceptable and an optimal fit, respectively [37], and RMSEA values of ≤0.10 indicate an acceptable fit [38].

Later, we tested whether the PSC-17-Y exhibits an invariant structure across gender and across time through longitudinal and multigroup measurement invariance analysis. In particular, 3 levels of invariance were tested: (1) *configural* (test whether all items load on the proposed factor), (2) *metric* (test whether item-factor loadings are similar across groups), and (3) *scalar* (test whether unstandardized item thresholds are similar across groups). In addition, within longitudinal measurement, invariance residuals covariances between the same item over time (eg, time 0 with time 1) were included. Thus, to indicate a significant decrement in fit when testing for measurement invariance, we used model comparison criteria of ΔCFI/ΔTFI ≥ 0.01 (ie, a decrease indicating the worst fit) [39] and ΔRMSEA ≥ 0.015 (ie, an increase indicating the worst fit) [40].

When there is scalar measurement invariance, the comparison of factor means across groups is permissible [41]. Consequently, we calculated gender differences. We also estimated the Cohen d index (standardized mean difference), which allows evaluating the effect size (ES) of the obtained differences [42].

The Cronbach *α* and the McDonald omega (*ω*) [43] were used to estimate the internal consistency of the PSC-17-Y since the McDonald *ω* is a better estimator of reliability than the Cronbach *α* [44].

Convergent and criterion validity was evaluated by calculating the correlation coefficients between the scores on the PSC-17-Y and different, well-established measures. Specifically, we tested the convergent validity with measures of distress (SEDS-S) and criterion validity with measures of well-being, QoL, and socioemotional skills (MHC-SF, KIDSCREEN-10 Index, SEHS-S). The Cohen criteria were used to estimate the ES of the correlations [42,45].

Finally, normative data for the PSC-17-Y were presented in the form of percentiles (75th and 90th). We also calculated the cut-off point of 15 for OVR, 5 for INT symptoms, 7 for EXT symptoms, and 7 for ATT, as proposed by Gardner et al [16,17], because these scores have not received evidence-based support in Spanish adolescents.

As the sample size determination for psychometric validation studies lacks clear recommendations [46], we determined the required sample size by allocating several observations 5-10 times greater than the variables [47]. Accordingly, the sample size needed ranged between 85 and 170 participants based on the number of items in the PSC-17-Y. Furthermore, according to the subject-to-item ratio method, a sample size of ≥1000 to perform exploratory factor analysis (EFA) or CFA would be excellent [46].

## Results

### Confirmatory Factor Analysis and Measurement Invariance

Findings of CFA and measurement invariance over time and across gender groups are summarized in Table 1. Since item 13 (“Me cuesta mucho cansarme”/“Acts as if driven by a motor”) had a low loading at the ATT subscale (0.127), we also tested the 16-item model, which slightly improved the fit. An optimal fit index was observed for both the 17- and 16-item models, with loadings from 0.348 (item 5) to 0.858 (item 6) and from 0.346 (item 5) to 0.859 (item 6), respectively. Longitudinal and multigroup measurement invariance was found for the 17- and 16-item models, which means that the structure, loadings, and intercepts of the PSC-17-Y were invariant over time and across gender groups. Although the 16-item version was slightly stronger than the 17-item version, we concluded that the minimal improvement in accuracy was less important than being able to use the 17-item model, which is the internationally recognized version and facilitates comparisons with other studies. For this reason, subsequent analyses were performed with a 17-item version.

**Table 1:** Goodness of fit for baseline models and measurement invariance of the 3-factor solution.

Number		Items, n	Overall goodness of fit				Comparative goodness of fit			
			*χ*^2^^a^ (*df*)	CFI^b^	TLI^c^	RMSEA^d^ (90% CI)	Model comparison	ΔCFI	ΔTLI	ΔRMSEA
**Baseline model**										
	1	17	1462.63 (116)	0.961	0.954	0.046 (0.044-0.048)	—^e^	—	—	—
	2	16	1105.35 (101)	0.970	0.965	0.043 (0.041-0.045)	1 vs 2	0.009	0.010	–0.003
**Longitudinal measurement configural invariance**										
	3	17	2814.41 (496)	0.956	0.950	0.029 (0 .028-0.030)	—	—	—	—
	4	16	1957.05 (433)	0.970	0.966	0.025 (0 .024-0.027)	—	—	—	—
**Longitudinal measurement metric invariance**										
	5	17	2395.55 (505)	0.964	0.960	0.026 (0 .025-0.027)	3 vs 5	0.008	0.010	–0.003
	6	16	1845.45 (443)	0.973	0.969	0.024 (0 .023-0.025)	4 vs 6	0.003	0.003	–0.001
**Longitudinal measurement scalar invariance**										
	7	17	2538.15 (522)	0.961	0.958	0.027 (0 .026-0.028)	5 vs 7	–0.003	–0.002	0.001
	8	16	1969.06 (459)	0.970	0.968	0.025 (0 .024-0.026)	6 vs 8	–0.003	–0.001	0.001
**Gender** **configural** **invariance**										
	9	17	2573.15 (990)	0.968	0.964	0.024 (0 .023-0.025)	—	—	—	—
	10	16	2110.28 (866)	0.974	0.971	0.023 (0 .022-0.024)	—	—	—	—
**Gender** **metric** **invariance**										
	11	17	2777.51 (1018)	0.965	0.961	0.025 (0 .024-0.026)	9 vs 11	–0.003	–0.003	0.001
	12	16	2342.64 (892)	0.970	0.967	0.024 (0 .023-0.026)	10 vs 12	–0.004	–0.004	–0.001
**Gender** **scalar** **invariance**										
	13	17	3068.45 (1046)	0.959	0.956	0.027 (0 .026-0.028)	11 vs 13	–0.006	–0.005	0.002
	14	16	2460.40 (918)	0.968	0.966	0.025 (0 .024-0.026)	12 vs 14	–0.002	–0.001	0.001

^a^*P*<.001.

^b^CFI: comparative fit index.

^c^TLI: Tucker-Lewis index.

^d^RMSEA: root-mean-square error of approximation.

^e^Not applicable.

The correlation matrix of the items can be seen in Table 2.

As can be seen in Tables 3 and 4, the reliability coefficients for all measures ranged from .64 to .76 (McDonald *ω*). Regarding gender differences among total scores, females showed significantly higher scores on general social-emotional distress (d=0.34) and INT (d=0.42) scales than males, with a small-to-medium ES. Concerning the differences in the EXT scale, males showed more symptoms of EXT problems than females, with a small ES (d=0.12). Although the scores for well-being and socioemotional competencies were lower in females than in males, the ESs were low (d ranged from 0.08 to 0.15). Finally, males showed higher HRQoL levels than females, with an ES of 0.35.

According to Table 5, the intercorrelation between the PSC-17-Y subscales was moderate, indicating that this measure is composed of 3 differentiated and mutually associated factors. Regarding the relationship between PSC-17-Y subscales and the remaining measures, the highest positive correlations were between the INT subscale and the measure of distress (large ES), while the association of the ATT and EXT subscales with distress was medium. Similarly, the correlation was higher (and negative) between the INT subscale and the HRQoL (large ES) than between different types of well-being and socioemotional competencies (moderate to large). However, the correlation between ATT and measures of well-being, socioemotional competencies, and the HRQoL was significant and negative, with a small-to-medium magnitude. Finally, the EXT subscale presented a small-to-medium association with the different positive measures.

**Table 2:** Correlation matrix of the items.

Items	1	2	3	4	5	6	7	8	9	10	11	12	13	14	15	16
1	—^a^	—	—	—	—	—	—	—	—	—	—	—	—	—	—	—
2	0.15	—	—	—	—	—	—	—	—	—	—	—	—	—	—	—
3	0.28	0.33	—	—	—	—	—	—	—	—	—	—	—	—	—	—
4	0.14	0.16	0.19	—	—	—	—	—	—	—	—	—	—	—	—	—
5	0.15	0.10	0.15	0.32	—	—	—	—	—	—	—	—	—	—	—	—
6	0.10	0.73	0.33	0.25	0.17	—	—	—	—	—	—	—	—	—	—	—
7	0.29	0.38	0.49	0.20	0.20	0.41	—	—	—	—	—	—	—	—	—	—
8	0.21	0.39	0.28	0.24	0.16	0.36	0.32	—	—	—	—	—	—	—	—	—
9	0.13	0.52	0.27	0.16	0.08	0.56	0.32	0.33	—	—	—	—	—	—	—	—
10	0.18	0.30	0.25	0.36	0.22	0.32	0.30	0.35	0.31	—	—	—	—	—	—	—
11	0.13	0.58	0.25	0.18	0.155	0.60	0.35	0.28	0.45	0.28	—	—	—	—	—	—
12	0.31	0.30	0.40	0.29	0.227	0.32	0.41	0.44	0.23	0.38	0.27	—	—	—	—	—
13	0.22	–0.04	0.05	0.07	0.105	0.06	0.06	0.06	–0.01	0.05	–0.01	0.16	—	—	—	—
14	0.26	0.24	0.34	0.31	0.256	0.24	0.33	0.41	0.20	0.41	0.19	0.48	0.14	—	—	—
15	0.13	0.42	0.20	0.07	0.026	0.41	0.30	0.23	0.47	0.18	0.36	0.17	0.07	0.14	—	—
16	.15	.24	.24	.29	.174	.28	.25	.37	.13	.34	.16	.43	.11	.42	.07	—
17	0.33	0.31	0.576	0.178	0.187	0.34	0.69	0.33	0.27	0.28	0.28	0.44	0.07	0.37	0.26	0.30

^a^Not applicable.

**Table 3:** Descriptive analysis for males and females and reliability coefficients of the PSC-17-Y^a^.

PSC-17-Y Subscales	Cronbach *α* (95% CI)		Interitem correlations			McDonald *ω* (95% CI)		Score, mean (SD)		Effect size (d)
	Female	Male	Mean	Min	Max	Female	Male	Female (x)	Male (y)	d = x – y (*P* value)
ATT^b^	.61 (0.59-0.63)	.63 (0.61-0.65)	0.306	0.050	0.686	.65 (0.63-0.67)	.64 (0.62-0.66)	4.45 (1.98)	4.46 (2.07)	0.01 (.76)
INT^c^ symptoms	.76 (0.75-0.78)	.73 (0.71-0.74)	0.510	0.412	0.730	.76 (0.75-0.78)	.72 (0.70-0.74)	3.74 (2.37)	2.79 (2.17)	0.42 (<.001)
EXT^d^ symptoms	.65 (0.63-0.67)	.67 (0.65-0.68)	0.327	0.161	0.348	.66 (0.64-0.68)	.67 (0.65-0.69)	2.54 (2.07)	2.80 (2.15)	–0.12 (<.001)

^a^PSC-17-Y: short form of the Pediatric Symptom Checklist-Youth Self-Report.

^b^ATT: attention-deficit/hyperactivity disorder symptoms.

^c^INT: internalizing.

^d^EXT: externalizing.

**Table 4:** Descriptive analysis for males and females and reliability coefficients of study measures.

Measures	Cronbach *α* (95% CI)		McDonald *ω* (95% CI)		Score, mean (SD)		Effect size (d)
	Female	Male	Female	Male	Female (x)	Male (y)	d = x – y (*P* value)
Emotional	.80 (0.79-0.81)	.77 (0.75-0.78)	.82 (0.80- 0.83)	.79 (0.77-0.81)	13.84 (3.29)	14.31 (3.06)	–0.15 (<.001)
Social	.85 (0.84-0.86)	.83 (0.82-0.84)	.86 (0.85-0.86)	.83 (0.82-0.84)	19.67 (5.60)	20.36 (5.50)	–0.12 (<.001)
Psychological	.86 (0.86-0.87)	.86 (0.85-0.87)	.91 (0.85-0.87)	.86 (0.85-0.87)	28.04 (5.74)	28.51 (5.58)	–0.08 (.003)
HRQoL^a^	.86 (0.85-0.86)	.83 (0.82-0.84)	.86 (0.85-0.86)	.83 (0.82-0.84)	37.39 (7.36)	39.81 (6.44)	–0.35 (<.001)
Socioemotional skills	.91 (0.91-0.92)	.90 (0.90-0.91)	.91 (0.91-0.92)	.90 (0.90-0.91)	110.69 (14.72)	112.28 (14.04)	–0.12 (<.001)

^a^HRQoL: health-related quality of life.

**Table 5:** Correlations between the PSC-17-Y^a^, distress, and well-being measures.

PSC-17-Y Subscales – Measures	ATT^b^	INT^c^ symptoms	EXT^d^ symptoms	Distress	Emotional well-being	Social well-being	Psychological well-being	HRQoL^e^	Socioemotional skills
ATT	1	—^f^	—	—	—	—	—	—	—
INT symptoms	0.35^g^	1	—	—	—	—	—	—	—
EXT symptoms	0.46^g^	0.36^g^	1	—	—	—	—	—	—
Distress	0.35^g^	0.67^g^	0.35^g^	1	—	—	—	—	—
Emotional well-being	–0.19^g^	–0.51^g^	–0.24^g^	–0.44^g^	1	—	—	—	—
Social well-being	–0.21^g^	–0.46^g^	–0.26^g^	–0.38^g^	0.65^g^	1	—	—	—
Psychological well-being	–0.23^g^	–0.50^g^	–0.31^g^	–0.42^g^	0.70^g^	0.72^g^	1	—	—
HRQoL	–0.30^g^	–0.67^g^	–0.34^g^	–0.60^g^	0.66^g^	0.63^g^	0.72^g^	1	—
Socioemotional skills	–0.31^g^	–0.45^g^	–0.41^g^	–0.39^g^	0.60^g^	0.61^g^	0.70^g^	0.67^g^	1

^a^PSC-17-Y: short form of the Pediatric Symptom Checklist-Youth Self-Report.

^b^ATT: attention-deficit/hyperactivity disorder symptoms.

^c^INT: internalizing.

^d^EXT: externalizing.

^e^HRQoL: health-related quality of life.

^f^Not applicable.

^g^*P*<.001.

Concerning normative information for PSC-17-Y subscales, each subscale is scored by the sum of its items. The adolescent’s score on the scale can then be used to obtain the corresponding percentile score. The normative information for each of the 3 PSC-17-Y subscales and total scores for the whole sample are shown in Table 6. We also included percentile scores for PSC-17-Y OVR scores to facilitate international comparisons.

The values obtained in our study using the international cut-off point of 15 for OVR, 7 for ATT, 5 for INT symptoms, and 7 for EXT symptoms, as proposed by Gardner et al [24], were 20.7%, 15.1%, 29.7%, and 5.1%, respectively. The 90th percentile indicated that 11.6% of the sample scored above this cut-off point of 17 for OVR on the PSC-17-Y, 26.3% of the participants exceeded the 75th percentile for the PSC-17-Y total score. Regarding specific symptoms, 15.1%, 10.2%, and 10.6% of the participants scored above the 90th percentile on ATT, INT symptoms, and EXT symptoms, respectively. The data at the 75th percentile or quartile 1 indicated that 29.1%, 28.7%, and 32.0% of the participants exceeded the cut-off points for ATT, INT symptoms, and EXT symptoms, respectively.

**Table 6:** Normative information about PSC-17-Y^a^ scales for adolescents (percentile scores); N=5430 (boys and girls 12-18 years old).

Percentile	PSC-17-Y ATT^b^	PSC-17-Y INT^c^ symptoms	PSC-17-Y EXT^d^ symptoms	PSC-17-Y total^e^
1	0	0	0	0
5	1	0	0	3
10	2	1	0	4
15	2	1	0	5
20	3	1	1	6
25	3	1	1	7
30	3	2	1	8
35	4	2	2	8
40	4	2	2	9
45	4	3	2	10
50	5	3	2	10
55	5	3	3	11
60	5	4	3	12
65	5	4	3	12
70	5	4	4	13
75	6	5	4	14
80	6	5	4	15
85	7	6	5	16
90	7	7	6	17
95	8	8	7	19
99	9	10	8	23

^a^PSC-17-Y: short form of the Pediatric Symptom Checklist-Youth Self-Report.

^b^ATT: attention-deficit/hyperactivity disorder symptoms. Rating anchor: ATT=0-10 (5 items).

^c^INT: internalizing. Rating anchor: INT symptoms=0-10 (5 items).

^d^EXT: externalizing. Rating anchor: EXT symptoms=0-14 (7 items).

^e^Rating anchor: total score=0-34 (17 items).

## Discussion

### Principal Findings

This study aimed to delineate the psychometric properties of the PSC-17-Y in Spanish adolescents. As expected, this study found evidence of a 3-factor solution, as in the original English version, and also gave evidence of reliability and validity (structural, convergent, and criterion) to assess several psychopathology symptoms among adolescents. However, item 13 showed a lower loading (0.127) than the remaining items. This finding is consistent with Bergmann et al. [22], who validated the PSC-17-Y in English and reported a relatively low factor loading (0.233) for this item. Following the same logic as these authors, we maintained item 13 in the final set of items for the PSC-17-Y in Spanish, since it is important to keep the measure as simple as possible for respondents and clinicians to complete, score, and interpret and since the parent- and youth-reported short forms were identical except for this 1 item. We found that the inclusion of item 13 had a negligible impact on the psychometric properties of the PSC-17-Y in Spanish. As a result, we elected to add item 13 to the 16-item model and recommend a 17-item short form of the PSC-Y that uses the same 17 items on the same 3 subscales as the parent-reported PSC-17. Furthermore, we hypothesized that the problem with this item could be the wording and that future studies might review it to improve the saturation in the factor. The PSC-17-Y also showed an invariant structure across both genders, again consistent with Bergman et al [22].

Additionally, our study provided the first evidence, as far we know, for longitudinal invariance, indicating that the PSC-17-Y in Spanish adolescents is stable over time. This finding implies that it is reasonable to conclude that growth or development in observed scores over time can be attributed to actual development or changes in the construct under investigation, not measurement problems [48]. Further studies that replicate our study over more extended periods are, of course, needed.

Once gender and longitudinal invariance were tested, this study provided new evidence on gender-attributable differences in the PSC-Y scales. The gender differences found in this study were consistent with the overwhelming prior research establishing that females are more likely to express INT symptoms and males are more likely to express EXT symptoms [49-51]. Furthermore, the small ES found in these gender differences is also consistent with other ones, which highlighted the small magnitude of gender differences in INT problems among children and adolescents [52]. These findings suggest that gender differences should be considered when pediatric and mental health professionals interpret PSC-17-Y results. Furthermore, gender is a crucial variable in the relationship between INT symptoms and suicide among adolescents, increasing this risk in females [53].

Regarding reliability evidence, our study showed McDonald *ω* values between .64 and .76, which are slightly lower than those shown by previous studies. Gardner et al [16] reported high internal consistency (.79 for INT symptoms, .83 for EXT symptoms, and .83 for ATT), and in the same order, Bergmann et al [22], with 16 items, found consistency values of .81, .74, and .69, respectively. The lowest internal consistency value was ATT (.65 and .64 for females and males, respectively). The removal of item 13 could improve the internal consistency of the ATT subscale slightly to values of .70 and .71 for females and males, respectively but at the cost of losing the original 17-item structure of the questionnaire.

Concerning other sources of convergent and criterion validity evidence of the PSC-17-Y, all 3 subscales correlated positively with the measure of distress and negatively with well-being, HRQoL, and social-emotional competencies, indicating a higher correlation between PSC-17-Y INT problems and the remaining convergent and criterion validity measures. This finding is consistent with previous studies on the PSC-17-Y, such as Parker et al [23], who reported screening validity of the PSC-17-Y in terms of higher scores on the PSC ATT and INT subscales among youth with any lifetime mental health diagnosis, as well as that ATT and INT subscale scores (but not EXT) are significantly correlated with psychosis, depression, and anxiety disorders. Thus, both our study and Parker et al’s [23] provide support for the convergent and discriminant validity of the PSC-17-Y.

Finally, regarding percentile scores, score distributions showed a positive asymmetric distribution, but these normative data can help locate specific and general psychopathological problems among adolescents. Our rates would be suggestive of adolescents presenting scores compatible with mental health symptoms likely being in the clinical range. These data are equivalent to those reported in previous studies using different versions and cut-off scores for the PSC, PSC-17, and PSC-17-Y: 5%-25% of children were screened positive [15,16,54-56]. For example, a study using the 90th percentile score on the PSC found that 10.4% of children had problems on the OVR scale [54]. Additionally, the prevalence rates found in our study are consistent with wide international reviews on estimates of mental health prevalence among adolescents [1].

### Limitations

The absence of an equivalent, well-established measure of INT, EXT, and ADHD measures; the absence of data on the area under the curve at optimal cut-off points in this study; and the sample's representativeness because we recruited the sample from the southeast of Spain exclusively were limitations of this study. In addition, there were only 2 waves of assessment in a short period of time (7 months), so it is recommended that future studies replicate the longitudinal invariance findings over longer intervals.

### Conclusion

This study showed that the PSC-17-Y is a useful, reliable, and valid ultrabrief screening measure for detecting mental health problems in adolescents and can be administered over the internet. More specifically, this study provided evidence of the reliability and validity (structural and convergent-discriminant) of the Spanish version of the PSC-17-Y for adolescents.

Finally, these findings are significant for the scientific community. Therefore, this work has allowed us to extend the evidence of the validity of the PSC-17-Y to another language and country (Spain) in a large sample of adolescents, where scores were invariant over time irrespective of gender. This is a requirement that few instruments meet or for which evidence has been provided. All of this supports the reliability of the PSC-17-Y’s assessments and its use in clinical contexts, such as monitoring the development of symptomatology. The fact that the PSC-17-Y is a tool that is easy to administer is another support for its use in clinical contexts.

Having instruments such as the PSC-17-Y with established reliability meets an especially important need during COVID-19 times, which have been characterized by an increase in mental health problems among children and adolescents [3-5] and a possibly greater need for case identification and outcome measurement.

Lastly, the results also support the use of the PSC-17-Y in longitudinal research, for example, for the study of the temporal trajectories of psychopathology in children, facilitating, among other things, reliability in the evaluation of the effectiveness of treatments. In addition to its usefulness in research, the PSC-17-Y is an instrument with applicability in the clinical setting, specifically in both primary care and specialty mental health units, as a screening tool for mental health problems in children and adolescents that is valid for monitoring changes in functioning over time.

## Abbreviations

ADHD: attention deficit hyperactivity disorder
ATT: attention deficit hyperactivity disorder symptoms
CFA: confirmatory factor analysis
CFI: comparative fit index
ES: effect size
EXT: externalizing
HRQoL: health-related quality of life
INT: internalizing
MHC-SF: Mental Health Continuum-Short Form
OVR: overall
PSC-17-Y: Short Form of the Pediatric Symptom Checklist-Youth Self-Report
PSC-17: Pediatric Symptom Checklist-Parent Version
PSC: Pediatric Symptom Checklist
RMSEA: root-mean-square error of approximation
SEDS-S: Social-Emotional Distress Survey-Secondary
SEHS-S: Social-Emotional Health Survey-Secondary
TLI: Tucker-Lewis index
WLSMV: weighted least squares means and variance adjusted
